# Expression of the relaxin family peptide 4 receptor by enterochromaffin cells of the mouse large intestine

**DOI:** 10.1007/s00441-022-03635-8

**Published:** 2022-05-21

**Authors:** Ada Koo, Ruslan V. Pustovit, Orla R. M. Woodward, Jo E. Lewis, Fiona M. Gribble, Mohammed Akhter Hossain, Frank Reimann, John B. Furness

**Affiliations:** 1 Department of Anatomy & Physiology, University of Melbourne, Parkville, VIC 3010 Australia; 2 Florey Institute of Neuroscience and Mental Health, Parkville, VIC 3010 Australia; 3 Wellcome Trust-MRC Institute of Metabolic Science-Metabolic Research Laboratories, University of Cambridge, Cambridge, CB2 OQQ UK; 4 Department of Biochemistry and Pharmacology, University of Melbourne, Parkville, VIC 3010 Australia

**Keywords:** INSL5, 5-HT, Enteroendocrine cells, Enteric nervous system, Colonic reflexes

## Abstract

The gastrointestinal hormone, insulin-like peptide 5 (INSL5), is found in large intestinal enteroendocrine cells (EEC). One of its functions is to stimulate nerve circuits that increase propulsive activity of the colon through its receptor, the relaxin family peptide 4 receptor (RXFP4). To investigate the mechanisms that link INSL5 to stimulation of propulsion, we have determined the localisation of cells expressing *Rxfp4* in the mouse colon, using a reporter mouse to locate cells expressing the gene. The fluorescent signal indicating the location of *Rxfp4* expression was in EEC, the greatest overlap of *Rxfp4*-dependent labelling being with cells containing 5-HT. In fact, > 90% of 5-HT cells were positive for *Rxfp4* labelling. A small proportion of cells with *Rxfp4*-dependent labelling was 5-HT-negative, 11–15% in the distal colon and rectum, and 35% in the proximal colon. Of these, some were identified as L-cells by immunoreactivity for oxyntomodulin. *Rxfp4*-dependent fluorescence was also found in a sparse population of nerve endings, where it was colocalised with CGRP. We used the RXFP4 agonist, INSL5-A13, to activate the receptor and probe the role of the 5-HT cells in which it is expressed. INSL5-A13 administered by i.p. injection to conscious mice caused an increase in colorectal propulsion that was antagonised by the 5-HT_3_ receptor blocker, alosetron, also given i.p. We conclude that stimuli that excite INSL5-containing colonic L-cells release INSL5 that, through RXFP4, excites 5-HT release from neighbouring endocrine cells, which in turn acts on 5-HT_3_ receptors of enteric sensory neurons to elicit propulsive reflexes.

## Introduction

Amongst gut endocrine cell products, insulin-like peptide 5 (INSL5) is confined to the distal large intestine, where it occurs in L-type enteroendocrine cells (EEC) that also contain glucagon-like peptide 1 (GLP-1) and peptide YY (PYY), both of these being costored with INSL5 in the same secretory vesicles of these EEC (Grosse et al. 2014; Billing et al. 2018; Vahkal et al. 2021). The administration of an INSL5 mimetic and stimulation of hormone release from colonic L-cells using DREADD technology both cause defecation, but there is no evidence that either GLP-1 or PYY is involved in defecation control (Diwakarla et al. 2020; Lewis et al. 2020; Pustovit et al. 2021). On the other hand, these peptides, GLP-1 and PYY, have roles in slowing gastric and upper intestinal transit (Lin et al. 1996; Holst 2007). The INSL5 mimetic had no effect in mice in which the receptor for INSL5, the relaxin family peptide 4 receptor (RXFP4), was knocked out (Diwakarla et al. 2020), implying that RXFP4 is downstream of the L-cell release of INSL5. The L-cells express receptors for microbial products, including free fatty acid receptor (FFAR) 2 and OLFR78 (Karaki et al. 2006, 2008; Husted et al. 2017; Billing et al. 2019), and instillation of a short-chain fatty acid mixture into the colon accelerates colonic emptying (Yajima 1985; Fukumoto et al. 2003). Acceleration of colonic propulsion by SCFAs in mice was inhibited by the RXFP4 receptor antagonist, INSL5-A13NR, implying that INSL5 has a physiological role to stimulate propulsion (Pustovit et al. 2021). There is also likely to be an involvement of 5-HT, acting through 5-HT_3_ receptors, because enhanced propulsion caused by SCFAs or by DREADD-mediated stimulation of L-cells was inhibited by 5-HT_3_ receptor antagonists (Fukumoto et al. 2003; Lewis et al. 2020).

A feasible interpretation of these results is that INSL5 released from L-cells acts on RXFP4 of adjacent enterochromaffin cells, causing release of 5-HT that stimulates the enteric nervous system to evoke propulsive reflexes (Pustovit et al. 2021). Supporting this interpretation, a recent study has located *Rxfp4*-dependent fluorescence to EEC of the mouse colon, 65% of these also expressing 5-HT (Lewis et al. 2021). The 5-HT-containing enteroendocrine cells in the mouse colon have a variety of shapes that can be best revealed in thick sections (Koo et al. 2021; Kuramoto et al. 2021). Amongst these are enterochromaffin cells with basal processes as long as several 100 µm, some of which form close relationships with L cells and cells with short or no basal processes (Koo et al. 2021).

In the current study, we have utilised an *Rxfp4*-dependent reporter mouse (Lewis et al. 2021) to investigate which of the types of 5-HT-containing EEC express *Rxfp4* throughout the mouse colon, and we have also investigated whether the stimulation of colonic propulsion using an RXFP4-specific agonist is inhibited by a 5-HT_3_ receptor blocker.

## Methods

### Tissue preparation

Tissue was harvested from RXFP4^EYFP^ mice that were generated by crossing *Rxfp4*-Cre mice with GFP-based fxSTOPfx reporter mice (Lewis et al. 2021). Four female RXFP4^EYFP^ mice were anesthetised and perfused through the heart with 4% paraformaldehyde (PFA). The large intestine, from the caecum to the internal anal sphincter, was removed and placed in the same fixative at 4 °C overnight. Fixative was removed by 5 washes in phosphate-buffered saline (PBS; 0.15 M NaCl in 0.01 M sodium phosphate buffer, pH7.2). Fixed large intestine tissues from four female Rxfp4-Cre:Het Rosa26-GCaMP3:Hom mice, 8–10 weeks old, stored in PBS-sucrose azide (0.1% w/v sodium azide and 30% w/v sucrose in PBS) on cool pack, were transferred from the Cambridge, UK, to the Melbourne, Australia, laboratories. Tissues were transferred to a 1:1 ratio of PBS-sucrose azide and OCT compound, and then, sections of proximal colon, distal colon, and rectum were embedded in 100% OCT compound and frozen in isopentane cooled with liquid nitrogen.

### Immuohistochemistry

Sections of 60 µm thickness were cut using a cryostat and placed in PBS. Tissues were blocked in normal horse serum (10% v/v in PBS with 1% Triton X-100) for 1 h at room temperature and then incubated with a mixture of primary antibodies (Table 1) for 3 nights at 4 °C. Sections were washed three times with PBS, 15 min each, followed by incubation with a mixture of secondary antibodies overnight at 4 °C. Sections were washed twice with PBS, 10 min each, and quenched with quenching buffer (5 mM copper sulphate and 50 mM ammonium acetate, pH5.0) for 1 h at room temperature. Sections were washed once with PBS and twice with distilled water, 5 min each, followed by an incubation with Hoechst 33,258 (10 µg/mL; Sigma-Aldrich, Sydney, NSW, Australia) for 45 min at room temperature. Sections were washed 3 times with distilled water, 5 min each, and then mounted on microscope slides in nonfluorescent mounting medium (Dako, Carpinteria, CA, USA).

**Table 1:** Primary and secondary antibodies used and their dilutions

	**Target**	**Catalogue number**	**Source**	**Species**	**Dilution**	**RRID**
**Primary**	GFP	Ab13970	Abcam, Melbourne, Australia	Chicken	1:5000	AB_300798
	5-HT	20,079	ImmunoStar, Hudson, Wi, USA	Goat	1:5000	AB_572262
	Oxyntomodulin	AB-323-AO010	Ansh Labs, Webster, Tx, USA	Mouse	1:1000	―
	CGRP	T4032	Peninsula Labs, Santa Cruz, Ca, USA	Rabbit	1:500	AB_518147
**Secondary**	Chicken IgG	703–545-155, Alexa Fluor® 488	Jackson ImmunoResearch Lab, West Grove, Pa, USA	Donkey	1:500	AB_2340375
	Goat IgG	A21432, Alexa Fluor® 555	Thermo Fisher Scientific, Scoresby, Australia	Donkey	1:800	AB_2535853
	Mouse IgG	A31571, Alexa Fluor® 647	Molecular Probes, Eugene, Or, USA	Donkey	1:2000	AB_162542
	Rabbit IgG	A32795, Alexa Fluor® 647	Thermo Fisher Scientific	Donkey	1:1000	AB_2762835

### Image acquisition and analysis

Images were captured using a super-resolution confocal microscope (LSM880 Airyscan Fast, Carl Zeiss, Sydney, NSW, Australia) using a 20 × air objective or 63 × oil objective. Captured images were deconvoluted using Airyscan Processing in Zeiss Zen (black edition) software prior to analysis. Brightness and contrast were adjusted using Fiji ImageJ (https://imagej.nih.gov/ij/), and then, cells were selected based on immunoreactivity and intensity and were exported for cell count analysis. Approximately, 100 cells were counted for each region from each of the 4 animals. A cell was considered immunopositive when intensity was greater than background mean plus two standard deviations. Example images were converted to RGB colour before exporting as TIFF files using Fiji ImageJ.

### Synthesis of RXFP4 agonist, INSL5-A13

INSL5-A13 was synthesized in house by our previoulsy published method (Patil et al. 2016). The A and B chains were each chemically assembled on solid-phase support. Following this, the disulphide bridges between the chains were formed in solution, and the two-chain compound was purified.

### In vivo studies

Mice were injected with vehicle, loperamide (1.0 mg/kg s.c.), or loperamide plus the 5-HT_3_ receptor antagonist, alosetron (1.0 mg/kg i.p.), and then 5 min later with the RXFP4 receptor antagonist, INSL5-A13 (6 ug/kg i.p.) or vehicle. After a further 20 min, colorectal propulsion was assessed using the bead expulsion test. Loperamide (Sigma-Aldrich, Sydney, NSW, Australia) was prepared in 1% Tween-80 in distilled water; alosetron HCl (Sigma-Aldrich) and INSL5-A13 were dissolved in distilled water. To measure bead expulsion, male mice, 20–30 g body weight, were briefly anaesthetized with 2% (v/v) isoflurane in 1 L/min O_2_ for a maximum of 15 s following induction with 4% isoflurane in 1L/min O_2_ (Pustovit et al. 2021). A 3-mm round bead was inserted 2 cm into the distal colon using a flexible, plastic rod. After bead insertion, mice were placed in individual clean cages. The time taken from bead insertion to bead expulsion was recorded. The maximum time allowed for bead expulsion was 30 min. This was a practical choice; in control, bead expulsion times were less than 100 s, and it was decided that 1800s was a sufficiently longer time to test for the effectiveness of loperamide to delay bead expulsion. If bead expulsion time was greater than 30 min, the mouse was left undisturbed in a quiet place, and the bead was recovered 5 or 10 min later. The time was recorded as 30 min and the data included. Agonist and antagonist experiments were conducted in the period 8:00 am to 1:00 pm. The same mice were used for successive tests, 1 week apart.

## Results

### Colocalisation of Rxfp4-GFP, 5-HT, and oxyntomodulin (OXM)

**Fig. 1:**
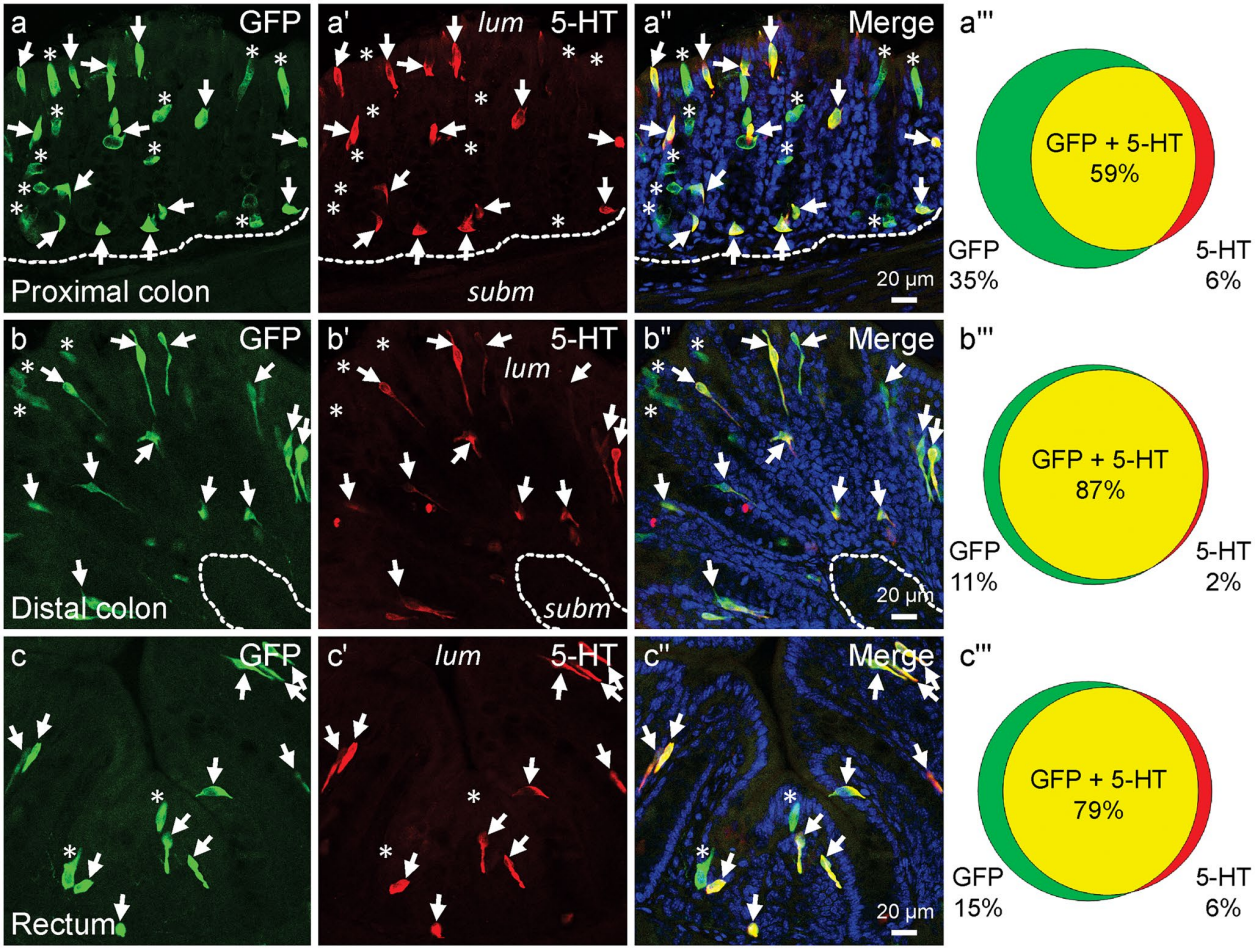
Coexpression of *Rxfp4*-GFP and 5-HT in EEC of the proximal colon (**a–a‴**), distal colon (**b–b‴**), and rectum (**c–c‴**). These transverse sections through the mucosa show EEC of various morphologies, including cells with long basal processes. The luminal (*lum*) and submucosal (*subm*) aspects of the mucosa are indicated in the 5-HT images. Nuclei are revealed by Hoechst 33,258 stain in the merged images. Double immunopositive cells for *Rxfp4*-GFP and 5-HT are marked by *arrows*, and cells expressed only *Rxfp4*-GFP are indicated by *asterisks*. Venn diagrams show the proportions of *Rxfp4*-GFP, 5-HT, or double immunoreactive cells of approximately 100 cells in each region from each of the 4 animals

A high degree of overlap of *Rxfp4*-dependent GFP and 5-HT was observed in EEC of the large intestine, with the greatest proportion of *Rxfp4*-GFP cells that expressed 5-HT being in the distal colon (Fig. 1). Of 5-HT cells, the great majority expressed *Rxfp4*-GFP; 93.5 ± 1.6% expressed *Rxfp4*-GFP in the proximal colon, 98.0 ± 1.5% in the distal colon, and 94.3 ± 2.9% in the rectum (Fig. 1). The colocalization encompassed all morphologies of 5-HT cells; in particular, 5-HT cells with long basal processes that have been recently described in the mouse large intestine (Kuramoto et al. 2021) expressed *Rxfp4*-GFP. It is notable that EEC with long processes were a greater proportion of 5-HT/ *Rxfp4* cells in the distal colon and rectum, compared to the proximal colon. This reflects relative abundances of 5-HT cells with different morphologies in the three regions (Koo et al. 2021).

In tissues from *Rxfp4*-GFP mice co-stained for GFP and 5-HT, 34.6 ± 7.1% of cells that were positive for either marker only expressed *Rxfp4*-GFP in the proximal colon, 10.9 ± 5.0% in the distal colon, and 15.5 ± 6.5% in the rectum (Fig. 1). When all staining was considered, in the proximal colon, coexpression of *Rxfp4*-GFP and 5-HT accounted for 59.0 ± 8.3% of all immunoreactive cells (Fig. 1a‴), and 87.0 ± 5.5% of all immunoreactive cells exhibited coexpression in the distal colon (Fig. 1b‴), and 78.7 ± 5.7% in the rectum (Fig. 1c‴).

**Fig. 2:**
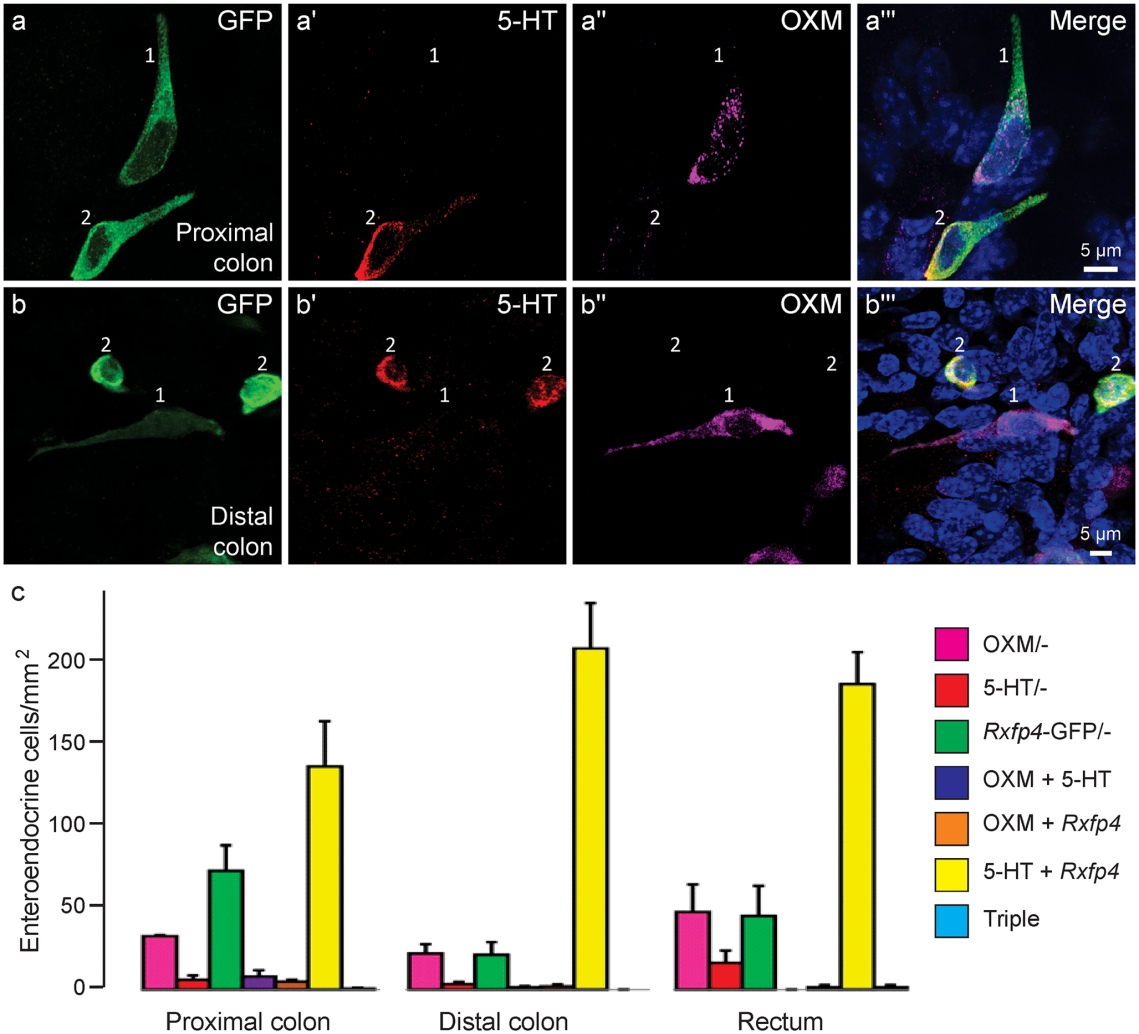
Colocalisation of *Rxfp4*-GFP, 5-HT, and OXM in EEC of the large intestine. Shown are examples of double-labelled *Rxfp4*-GFP and OXM cells (1) and double-labelled *Rxfp4*-GFP and 5-HT cells (2) in the proximal colon (**a–a‴**) and the distal colon (**b–b‴**). **c** Quantification of immuoreactive cells, from 4 mice, expressed as numbers of cells per total tissue area in mm^2^. OXM/ − indicates oxyntomodulin only (L cells without *Rxfp4*-GFP or 5-HT), etc. The most numerous cell type is 5-HT cells showing fluorescence for *Rxfp4*-GFP (yellow columns). Data are mean ± SEM

We next examined the expression of OXM, as a marker for L cells, in relation to *Rxfp4*-GFP and 5-HT-positive cells in the proximal colon, distal colon, and rectum. Interestingly, we observed scattered coexpression of OXM and *Rxfp4*-GFP amongst EEC (Fig. 2 a–b‴), which was more commonly found in proximal colon (4.8 ± 1.0 cells/mm^2^) than distal colon (2.0 ± 1.2 cells/mm^2^) and rectum (1.4 ± 1.4 cells/mm^2^). There were also some OXM and 5-HT double immunoreactive, but *Rxfp4*-GFP negative, cells in the proximal colon (7.9 ± 3.8 cells/mm^2^) and the distal colon (1.4 ± 0.8 cells/mm^2^) but none in the rectum. Amongst OXM, *Rxfp4*-GFP, and 5-HT-positive cells, the majority of immunopositive cells were those that coexpressed *Rxfp4*-GFP and 5-HT (Fig. 2c).

### Rxfp4-GFP-positive nerve fibres

**Fig. 3:**
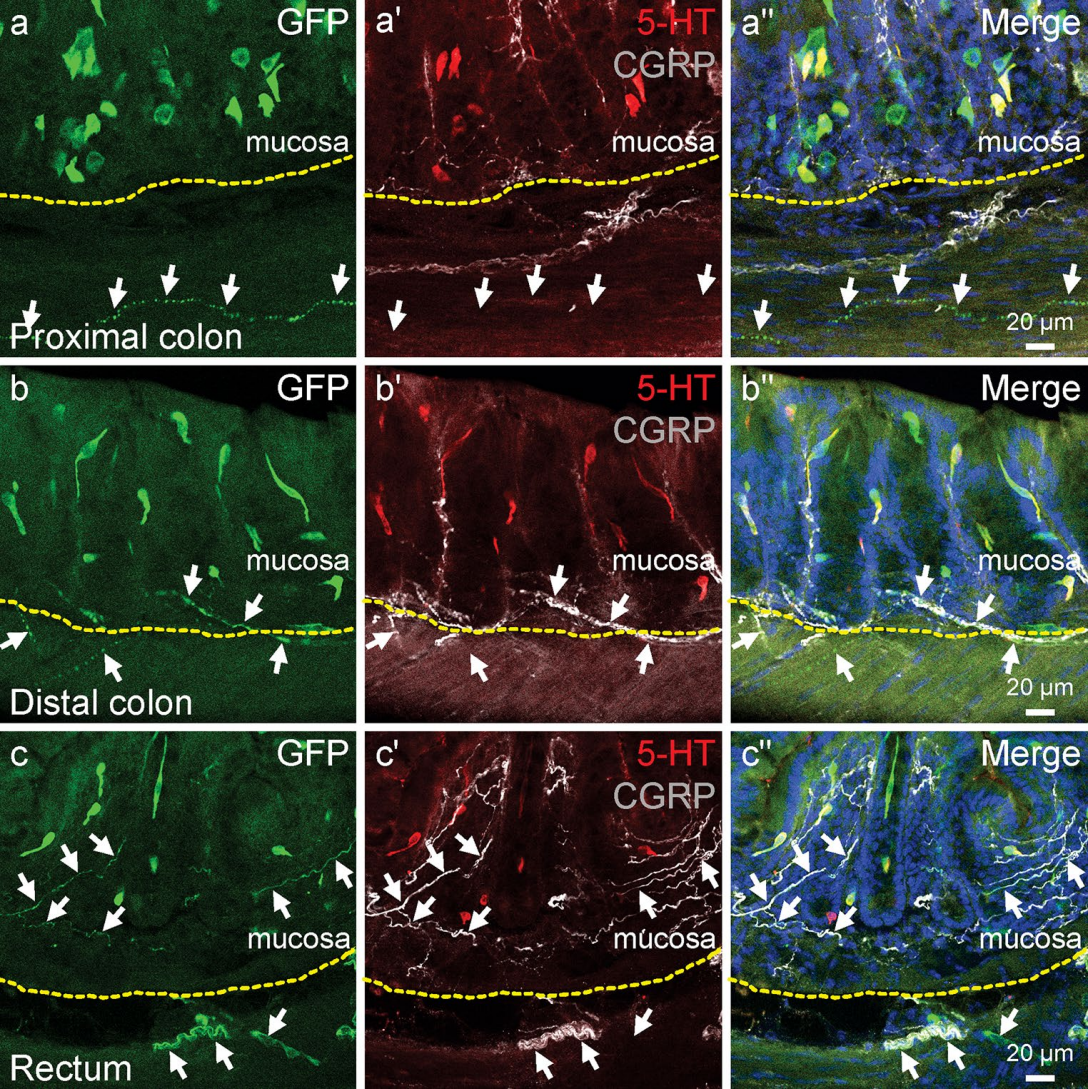
*Rxfp4*-GFP positive nerve fibres in the muscle layer and submucosa in the proximal colon (**a–a″**), distal colon (**b–b″**), and rectum (**c–c″**) and the relations of 5-HT and CGRP to *Rxfp4*-GFP. Many of the *Rxfp4*-GFP-positive fibres were CGRP immunoreactive. At this magnification, it is difficult to identify 5-HT immunoreactive nerve fibres. *Arrows* indicate *Rxfp4*-GFP-positive fibres

**Fig. 4:**
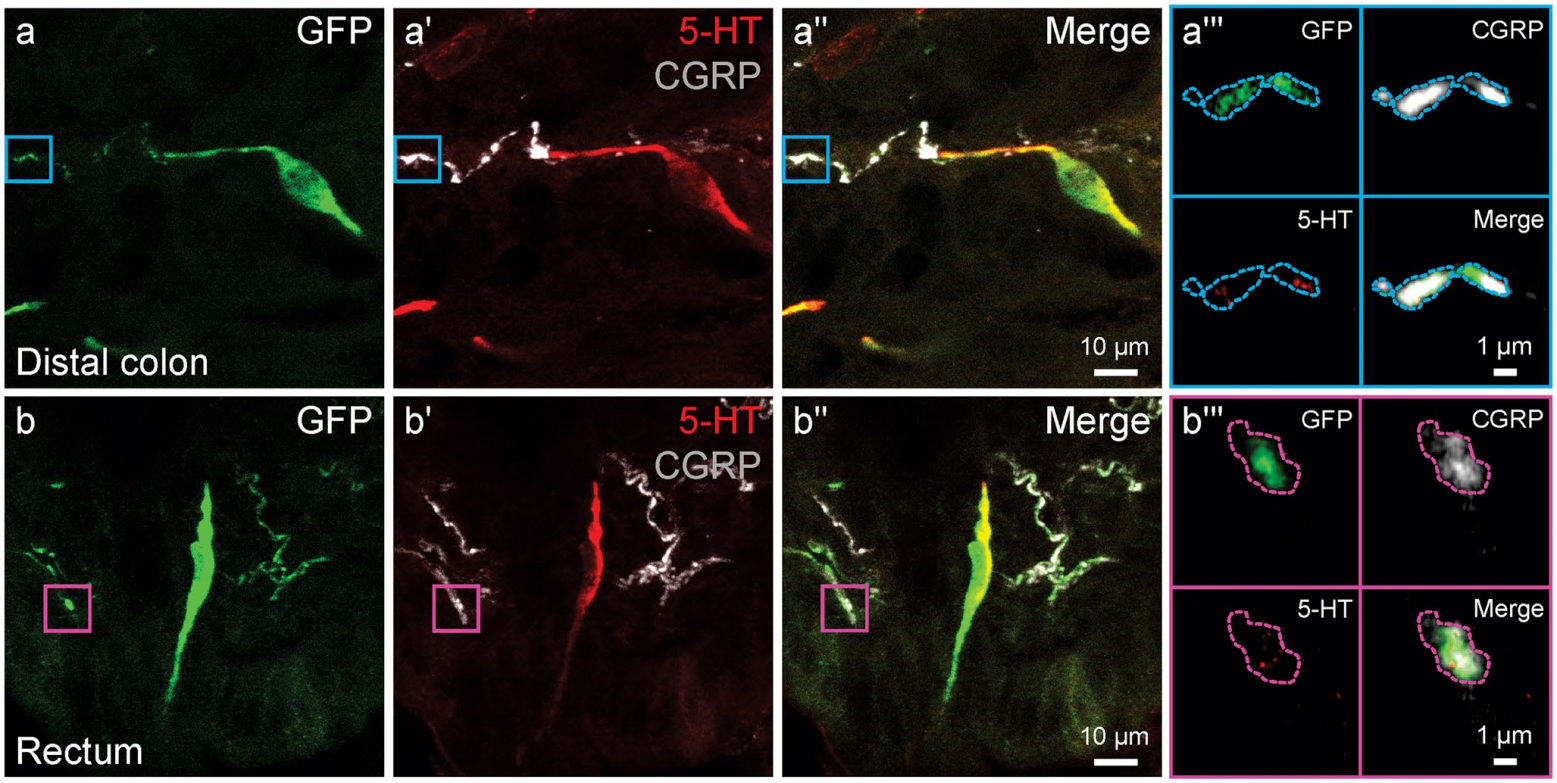
Colocalisation of *Rxfp4*-GFP, CGRP, and 5-HT in nerve fibre varicosities in the distal colon (**a–a″**) and rectum (**b–b″**). **a‴** and **b‴** Selected zoomed region of a–a″ and b–b″ with corresponding coloured box, showing overlap of *Rxfp4*-GFP and CGRP with 5-HT puncta within varicosities that are outlined by dotted lines. Note that the localisations of GFP, CGRP immunoreactivity, and 5-HT immunoreactivity within the varicosities are different

Nerve fibres with *Rxfp4*-GFP immunoreactivity were observed in all regions of the large intestine and were primarily found in the external muscle layers and submucosa (Fig. 3a–c). Triple staining for GFP, 5-HT, and CGRP was performed to further examine the relationship of *Rxfp4*-GFP-positive fibres and CGRP containing fibres in the mucosa. *Rxfp4*-GFP-positive fibres were not observed in the mucosa of the proximal colon; however, they were found in distal colon and rectum (Fig. 3b, c). The rectum was more densely innervated by *Rxfp4*-GFP and CGRP fibres than distal colon (Fig. 3c–c″). Higher-power super-resolution microscopy was performed to examine the possible colocalisation of *Rxfp4*-GFP and CGRP, which revealed double-labelled varicosities in the distal colon and rectum (Fig. 4). Interestingly, 5-HT immunoreactivity was also detected in some of the double-labelled varicosities and appeared to be stored in vesicles (Fig. 4a‴, b‴), unlike the diffused labelling of CGRP. The coexpression of *Rxfp4*-GFP and CGRP indicated that *Rxfp4*-GFP-postive fibres could be sensory nerve fibres orginating from the dorsal root ganglia, in which the expression of *Rxfp4* has been reported (Lewis et al. 2021).

### Effect of the 5-HT_3_ receptor antagonist, alosetron, on RXFP4 agonism

**Fig. 5:**
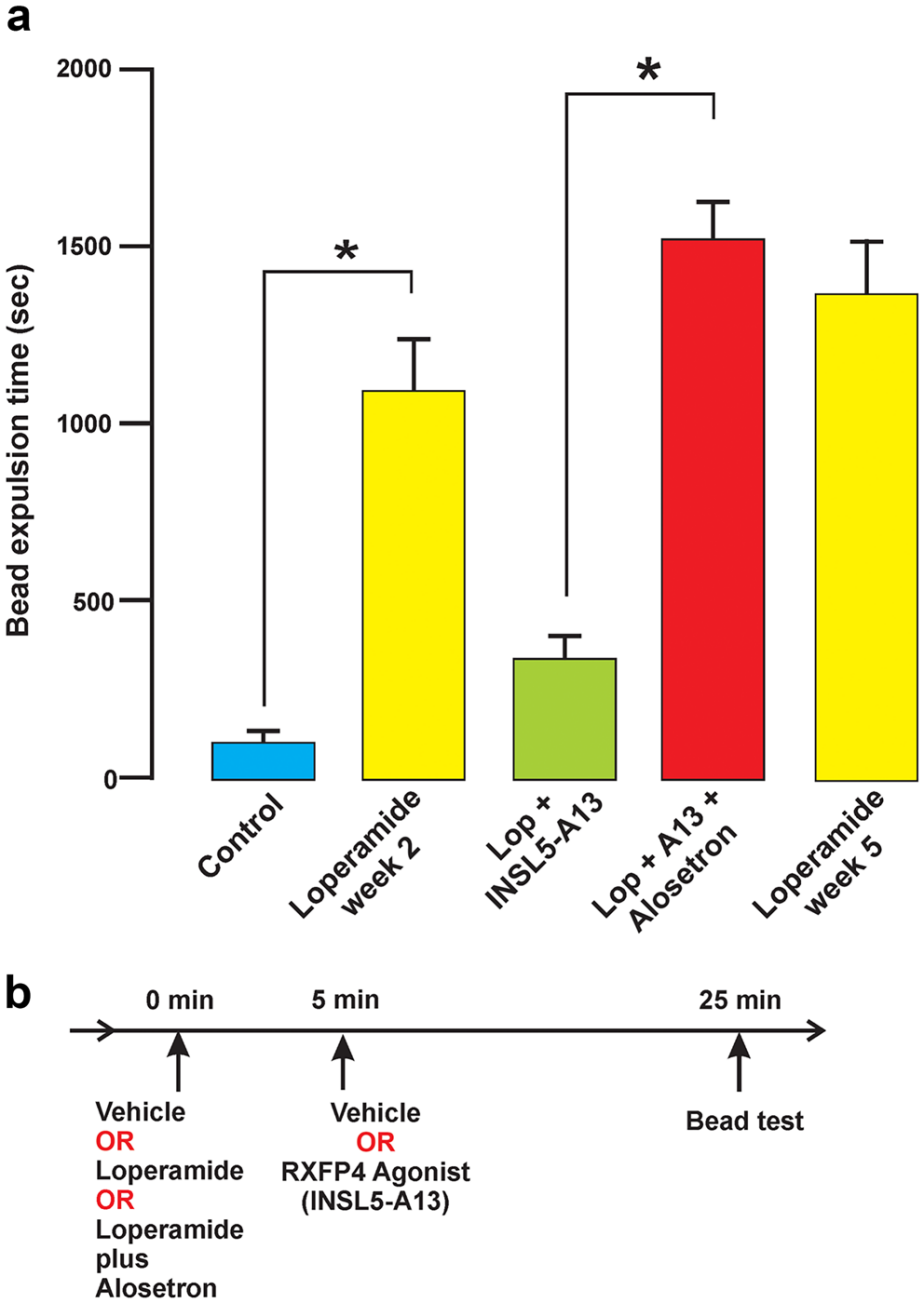
Reversal of the stimulation of colorectal propulsion with the RXFP4 agonist, INSL5-A13, by the 5-HT_3_ receptor antagonist, alosetron. **A** Bead expulsion times recorded from conscious mice. The same mice were treated with no drug in week 1 (blue column marked control), loperamide alone in week 2, loperamide plus INSL5-A13 in week 3, loperamide plus INSL5-A13 and alosetron in week 4, and loperamide alone in week 5. Loperamide significantly delayed bead expulsion compared to control (*p* $<$ 0.05). This effect was substantially reversed by INSL5-A13 (green column). Alosetron significantly delayed bead expulsion that had been accelerated by INSL5-A13 (loperamide/INSL5-A13 compared with loperamide + INSL5-A13 and alosetron, *p $<$ 0.05). **B** Timing of drug administration to mice. Lop, loperamide. Plotted are mean ± SEM, *n* = 9 mice

We used colorectal bead expulsion in conscious mice to investigate whether 5-HT is involved in the acceleration of colorectal propulsion that is evoked by stimulation of RXFP4. The same mice were investigated in successive weeks. In week 1, control bead expulsion times were determined, and in week 2, the slowing by loperamide (opiate agonist) was measured. Loperamide (1 mg/kg, s.c.) slowed bead expulsion times, determined 25 min after loperamide, approximately eightfold (Fig. 5). When the RXFP4 agonist, INSL5-A13 (6 µg/kg, i.p.), was given 5 min after loperamide, expulsion times measured 20 min later were reduced (Fig. 5). This acceleration of bead expulsion is due to action at RXFP4, as it is not observed in animals in which RXFP4 is knocked out (Diwakarla et al. 2020). The effect of INSL5-A13 to accelerate colonic bead expulsion was prevented by the 5-HT_3_ receptor antagonist, alosetron (1 mg/kg, i.p.), given at the same time as loperamide (Fig. 5, *p* < 0.05). In the week after (week 5), the loperamide effect was tested again because there are sometimes changes in sensitivity to opiate agonists. There was no difference between bead expulsion times with the two loperamide applications (yellow columns, Fig. 5).

## Discussion

The current work revealed that over 90% of 5-HT cells in the murine large intestine express *Rxfp4*, which implies that the 5-HT cells are downstream of the L cells that release the RXFP4 natural agonist, INSL5. In the mouse large intestine, there are two subtypes of L cells: those in the proximal colon express PYY, GLP-1, and neurotensin, but rarely INSL5 (L^Nts^ cells), and those in the distal colon express PYY, GLP-1, and INSL5 (L^Insl5^ cells) (Billing et al. 2019). In human, neurotensin is not expressed in the large intestine, but, like mouse, expression of *Insl5* is higher distally in the large intestine, and it is absent from the small intestine (Wang et al. 2020). When mice in which L cells of the distal colon (L^Insl5^ cells) that expressed a DREADD under the control of the *Insl5* promotor were stimulated with clozapine N-oxide (CNO; i.p.), there was an increase in defecation that was inhibited by a 5-HT_3_ receptor antagonist (Lewis et al. 2020). Colonic L cells express receptors for short-chain fatty acids (SCFA) (Karaki et al. 2006, 2008; Husted et al. 2017; Billing et al. 2019), and in a previous study, we showed that administration of a SCFA mix into the lumen of the large intestine caused an increase in colorectal bead expulsion and defecation that was blocked by an antagonist of the RXFP4 receptor (Pustovit et al. 2021). In the current study, we found a similar degree of antagonism of colorectal propulsion with the 5-HT_3_ antagonist, alosetron, when the RXFP4 agonist, INSL5-A13, was used to stimulate colorectal propulsion. These data confirm previous studies that enteric motility reflexes can be initiated through 5-HT_3_ receptors. The receptors are on the terminals of enteric intrinsic primary afferent neurons (IPANs) and are activated by 5-HT applied to the mucosa (Bertrand et al. 2000). The terminals of IPANs form a rich network of fibres beneath the mucosal epithelium where the 5-HT-containing endocrine cells are located (Furness et al. 1990, 2004).

INSL5-containing L cells of the mouse colon express functional receptors for a number of other GPCRs including receptors for bile acids, amino acids and peptones, angiotensin-II, vasopressin, and bombesin, all of which cause INSL5 release from EEC (Billing et al. 2018). Thus, like SCFAs, each of these is likely to increase the release of 5-HT indirectly through the INSL5/ RXFP4 system.

### Possible overlapping roles of colonic enterochromaffin (EC) cells

Enterochromaffin (5-HT-containing) cells in the large intestine, like L cells, also express microbial metabolite receptors, *Ffar2*, *Olfr78*, and *Olfr558*, as well as the bile acid receptor, *Gpbar1* (Lund et al. 2018; Billing et al. 2019). This implies that there are both indirect, via L cell release of INSL5, and direct effects of microbial metabolites on EC cells. Moreover, the mechanosensitive ion channel *Piezo2* is expressed by about 58 ± 5% of colonic EC cells (Alcaino et al. 2018), which is consistent with earlier observations that mechanical stimulation of the mucosa causes 5-HT release (Bülbring and Crema 1959; Grider et al. 1996). A higher proportion of EC cells expressed *Piezo2* in the distal compared to the proximal colon (Billing et al. 2019). Thus, the majority of 5-HT cells in the distal colon, where > 95% exhibit *Rxfp4*-dependent labelling, are predicted to respond to both mechanical distortion and SCFAs. In the distal colon, about 20% of EC cells have prominent long basal processes, and 58% have long or intermediate length basal processes (Kuramoto et al. 2021). Many of these cells must express *Rxfp4*. The long processes might be assumed to be associated with mechansensitivity, although EC cells in the small intestine, which express *Piezo2* and are mechanosensitive (Alcaino et al. 2018), do not have basal processes (Koo et al. 2021).

### RXFP4 nerve fibres

We observed a small population of nerve fibres positive for *Rxfp4*-GFP in the mucosa and adjacent submucosa that were immunoreactive for CGRP. We have not specifically investigated the origins of these fibres, but the majority of spinal afferent (dorsal root ganglion) fibres that supply the gastrointestinal tract in mice and other mammals are CGRP immunoreactive (Tan et al. 2010). Furthermore, *Rxfp4*-dependent fluorescence is observed in small diameter nerve cells, of the type that express CGRP, and in the dorsal root ganglia of mice (Lewis et al. 2021). *Rxfp4*-GFP labelling was not found in enteric neurons (Lewis et al. 2021), which are therefore deduced not to be a source of *Rxfp4*/CGRP nerve fibres in the colon.

## Concluding remarks: integrative roles of 5-HT cells

As discussed above, the 5-HT cells receive signals from L cells through INSL5 and its receptor, RXFP4, also express receptors for bacterial metabolites and secondary bile acids, and are mechanoreceptive. The L cells themselves have bacterial metabolite receptors. Thus, it appears that a range of stimuli, such as SCFAs, bile metabolites, and mechanical distortion, are integrated by the 5-HT-secreting EC cells, which may be a common pathway for different stimuli that influence colonic and rectal motility. Studies in which combinations of stimuli are applied may assist in unravelling how responses to different stimuli are integrated by the 5-HT cell population.
